# EXPERIENCES OF STAFF MEMBERS RELOCATING FROM A NURSING HOME TO AN INNOVATIVE LIVING ARRANGEMENT FOR OLDER ADULTS

**DOI:** 10.1093/geroni/igad104.0769

**Published:** 2023-12-21

**Authors:** Mara Brouwers, Bram Boer, Wim Groen, Hilde Verbeek

**Affiliations:** Maastricht university, Maastricht, Limburg, Netherlands; Maastricht University, Maastricht, Limburg, Netherlands; Amsterdam UMC, Amsterdam, Noord-Holland, Netherlands; Maastricht University, Maastricht, Limburg, Netherlands

## Abstract

More innovative living arrangements are developed within long-term care, leading to an increase in relocations. Relocating within long-term care is a long pending and intensive undertaking, especially for staff members. The combination of relocating and achieving a successful culture change might be a daunting task for staff members. Given the increase in the number of relocations and the fast development of innovative living arrangements, it is key to expand our knowledge concerning the impact such an undertaking has. The research question is therefore: ‘How is the relocation from a regular to an innovative living arrangement experienced by staff members?’ To answer this research question, 41 semi-structured interviews with staff members of several nursing homes undergoing a relocation were conducted. These nursing homes had to undergo a relocation to an innovative living arrangement. Findings indicate that a relocation process within long-term care is an intensive and stressful undertaking. Staff members often describe the relocation process as ‘stressful’, ‘chaotic’, and ‘intense’. Furthermore, although staff members are mostly positive about the new physical environment, they experience an array of barriers when trying to implement a new way of working. They define two key elements for successfully relocating and implementing a new way of working. First, clear communication and receiving sufficient information throughout the relocation process. Second, proper preparation for implementing a new way of working and coaching on the job. It is evident that relocating to an innovative living arrangement is a large undertaking that requires careful planning.

